# Humans have a basic physical and psychological need to move the body: Physical activity as a primary drive

**DOI:** 10.3389/fpsyg.2023.1134049

**Published:** 2023-04-11

**Authors:** Matthew A. Stults-Kolehmainen

**Affiliations:** ^1^Division of Digestive Health, Yale New Haven Hospital, New Haven, CT, United States; ^2^Department of Biobehavioral Sciences, Teachers College – Columbia University, New York, NY, United States

**Keywords:** drive, motivation, affectively charged motivation states, satiation, exercise, physical activity, non-exercise activity thermogenesis

## Abstract

Physical activity, while less necessary for survival in modern times, is still essential for thriving in life, and low levels of movement are related to numerous physical and mental health problems. However, we poorly understand why people move on a day-to-day basis and how to promote greater energy expenditure. Recently, there has been a turn to understand automatic processes with close examination of older theories of behavior. This has co-occurred with new developments in the study of non-exercise activity thermogenesis (NEAT). In this narrative review, it is hypothesized that psycho-physiological drive is important to understand movement in general and NEAT, specifically. Drive, in short, is a motivation state, characterized by arousal and felt tension, energizing the organism to acquire a basic need. Movement is a biological necessity, like food, water, and sleep, but varies across the lifespan and having the greatest impact before adolescence. Movement meets various criteria for a primary drive: (a) deprivation of it produces feelings of tension, such as an urge or craving, known as affectively-charged motivation states, and particularly the feelings of being antsy, restless, hyper or cooped up, (b) provision of the need quickly reduces tension - one can be satiated, and may even over-consume, (c) it can be provoked by qualities of the environment, (d) it is under homeostatic control, (e) there is an appetite (i.e., appetence) for movement but also aversion, and (f) it has a developmental time course. Evidence for drive has mainly come from children and populations with hyperkinetic disorders, such as those with anorexia nervosa, restless legs syndrome, and akathisia. It is also stimulated in conditions of deprivation, such as bed rest, quarantine, long flights, and physical restraint. It seems to be lacking in the hypokinetic disorders, such as depression and Parkinson’s. Thus, drive is associated with displeasure and negative reinforcement, subsuming it within the theory of hedonic drive, but it may fit better within new paradigms, such as the WANT model (Wants and Aversions for Neuromuscular Tasks). Recently developed measurement tools, such as the CRAVE scale, may permit the earnest investigation of movement drive, satiation, and motivation states in humans.

## Introduction

Movement is important for both physical and mental health, thriving in life and until recently, even survival. While exercise is widely recognized as improving physical fitness and cardiovascular health (Garber et al., 2011; Usdhhs, 2018; Bull et al., 2020), less recognized is the importance of bodily movement in general, which is necessary for proper circulation, tissue perfusion (i.e., oxygenation of tissues), metabolism, and many other functions (Greenleaf and Kozlowski, 1982; DeRoshia and Greenleaf, 1993). Those who move more frequently have less occurrence of blood clots (i.e., pulmonary embolisms, deep vein thromboses), frozen joints, cartilage degeneration, impaired digestion, metabolic disease and even skin problems (Lee et al., 2012; Musumeci et al., 2013; Crane et al., 2015; Pedersen and Saltin, 2015; Khmaladze et al., 2020). Under conditions of movement, weight is maintained in the face of increased or decreased calories (Levine et al., 1999, 2005; Donnelly et al., 2009). Movement is needed for proper growth, maturation and development of immune function (Vicente-Rodríguez, 2006). Mental health and even basic perception and psychological attention is facilitated with movement and degrades with lack of it (Zubek, 1964; Greenleaf and Kozlowski, 1982; Winget and Deroshia, 1986; Kim et al., 2012; Chekroud et al., 2018; de Sousa et al., 2018a,b; Parker et al., 2020; Pearce et al., 2022). Even when sleeping, a healthy body continues to move regularly, with about 135 total movements per session of sleep, of which 15.1 are major postural adjustments (Wilde-Frenz and Schulz, 1983). If indisposed, the body must still be moved frequently (e.g., by a nurse) to prevent pressure injuries (i.e., skin ulcers), additionally highlighting the importance of movement (Walton-Geer, 2009). Sudden declines in movement (i.e., outside of normal sleep and rest) are associated with precipitous worsening of health and accelerated aging (Greenleaf and Kozlowski, 1982; Winget and Deroshia, 1986). Engagement in a new and regular exercise routine results in immense health benefits that is dose-dependent, leading some to call it a “polypill” (Fiuza-Luces et al., 2013). Consequently, one might imagine that movement is as vital as food, water, air, sleep, shelter, and sexual activity – in other words, a basic or genuine need.

Movement falls on a spectrum of energy expenditure and movement intensity, ranging from sleep to vigorous activity (Rosenberger et al., 2019; Biddle, 2022). Furthermore, movement varies cyclically over a 24-h activity cycle (Rosenberger et al., 2019), typically higher when one is awake and peaking in the afternoon (McDonnell et al., 2022). Energy expenditure, *per se*, is largely not under volitional control, with the majority of energy being expended automatically through the resting metabolic rate (RMR) and the thermic effect of food (TEF; King et al., 2007). Substantial energy expenditure, however, is under volitional control, such as lifestyle and occupational physical activity and structured exercise. All physical activity not accounted for by exercise, RMR or TEF is deemed non-exercise activity thermogenesis (NEAT), which cannot be directly measured (Levine et al., 1999, 2005). Within NEAT falls incidental or spontaneous physical activity (SPA), such as fidgeting, pacing, and postural adjustments, which may also result in substantial energy expenditure (Garland et al., 2011). Some sedentary behavior common for our ancestors and in hunter gatherers of today, such as “static squatting,” results in enough muscular activity that it could be construed as a form of physical activity. This signifies the complexity of separating physically active and sedentary behaviors, the latter of which typically includes sitting (Raichlen et al., 2020; Higgins et al., 2022). For this hypothesis and theory paper, it is important to note that some volitional activity could also be characterized as “obligatory” (Garland et al., 2011) and instrumental (Stults-Kolehmainen et al., 2020a; see below).

Unfortunately, with modern times, physical activity and exercise have declined, and sedentarism has become dominant (Hyde et al., 2021; Ussery et al., 2021). Likewise, calories are abundant along with expanding waistlines and central adiposity (Fryar et al., 2021; Wong et al., 2022). The putative mechanism is that calories (e.g., particularly from highly palatable food) are highly reinforcing, while movement is less attractive. Reinforcement, however, has two forms, both positive (i.e., providing a pleasurable stimulus) and negative (i.e., taking away a negative stimulus). Movement as a positive reinforcer has been considered extensively (Cheval et al., 2018). Atypically has movement been considered as a negative reinforcer, such as taking away displeasure (Stults-Kolehmainen et al., 2020a). Drive theory contends that humans are motivated to extinguish the negative sensations that are usually produced through the deprivation of basic needs (Hull, 1943, 1952). Bodily movement has periodically been considered as a basic need (Bridges, 1936; Seward and Seward, 1937; Rowland, 2016). However, while instances of low movement are abundant, it’s uncommon in parlance to think of oneself as being *deprived* of movement. In fact, many may prefer it that way. Nevertheless, for some, there may be an obvious need to move, and a sense of deprivation when it is lacking. Furthermore, modern innovations have also produced situations where humans, who are otherwise active, become constrained, sometimes for long periods (e.g., academic lectures, flights, space travel, laboratory tasks, quarantine, various forms of confinement and sensory deprivation; Galton, 1885; Zubek, 1964; Zuckerman et al., 1968; Reardon et al., 2008; Shalev, 2008; Morgan et al., 2013; Seli et al., 2014; Bouwens et al., 2018; Blacutt et al., 2021; Stults-Kolehmainen et al., 2021a; Filgueiras and Stults-Kolehmainen, 2022). If movement is a basic need, there should be a sense of deprivation in these instances. Given these observations, it seems worthwhile to consider whether human movement has drive-like qualities, if it might even be considered a primary drive, and if so, what might be implicated for theories of physical activity behavior. The goal of this hypothesis and theory paper is to explore these issues in both a historical and modern context, drawing on a wide variety of literature. The goal is not to conduct a systematic review, particularly given the difficulties of searching for the term “drive,” and because there is no research question, *per se*.

## Discussion

### Theories of physical activity behavior

Human movement as a behavior remains an enigma. Dominant theories of exercise and physical activity behavior have largely failed to explain human movement in its totality, largely leaving the concept of daily energy expenditure to the physiologist (Garland et al., 2011). Theories of motivation have typically focused on motives and self-determination, as well other cognitive-oriented constructs (Biddle, 1995; Ryan and Deci, 2007; Stults-Kolehmainen et al., 2013a,b; Ekkekakis and Brand, 2021). There has been a recent resurgence of interest in older psychological theories in the construction of dual process theories of motivation (Conroy and Berry, 2017; Brand and Ekkekakis, 2018), which posit both automatic and deliberative components to motivation. For instance, ART theory has resurrected Lewin’s ideas of driving and restraining forces (Marrow, 1977; Brand and Ekkekakis, 2018), yet, attempts to reconcile these inputs on behavior are not unique. Butt (1976) commented that, “motivation may be seen as evolving from two major sources: a biologically-based fund of energy, and all secondary or environmental influences, each with positive and negative pulls.” Alderman (1974), took a more comprehensive view, stating, “motivated behavior is the sum total of instincts and needs, motives and drives, conscious and unconscious forces, and a function of what one expects to gain from participation in sport,” which Feige (1976) categorized hierarchically to explain physical activity motivation. The ideas of action impulse and urge have also reemerged, though they remain very poorly defined across the literature (Gardner, 2015; Brand and Ekkekakis, 2018; Rebar et al., 2018; Stults-Kolehmainen et al., 2020a, 2022a; Ekkekakis and Brand, 2021; Williams, 2023).

With attention now placed on older theories and constructs and rectifying them with newer ones, it begs the question of the relevance of some of the oldest theories of motivation, Drive Theory and the related Drive Reduction Theory (Hull, 1943, 1952). Drive theory posits that humans are motivated primarily to reduce tension and arousal, typically occurring from the deprivation of basic needs (Allen et al., 2017). With the dawning of behaviorism and cognitivism, this idea has been largely ignored, relegated or simply forgotten (Skinner and Morse, 1958). Early sport and exercise psychologists were disappointed with the ability of Drive Theory to predict the effects of arousal, and in particular anxiety, on performance of complex motor tasks, and they recommended the abandonment of the concept (Martens, 1971, 1974; Landers, 1980; Tenenbaum and Bar-Eli, 1995). These researchers had little interest in applying the concept to general movement and physical activity behavior. Those that were interested in exercise behavior were turned off by the idea of “people as machines” (Biddle, 1995).

However, the idea has persisted in the work of exercise physiologists within the notion of *biological control* (Rowland, 1998, 1999, 2016). In the current exercise psychology literature, “drive” is usually used in the context of basic psychological needs, as defined by Self-Determination Theory (Rhodes et al., 2019), nervous system activity (i.e., sympathetic and parasympathetic drive; Greenwood and Fleshner, 2019), as well as use in expressions of being “driven” (Frijda, 2016; Lichtenstein and Jensen, 2016). In fields outside of exercise science, there has been an earnest re-examination of the concept (Allen et al., 2017) because, while drive has been roundly criticized by some scholars, no meaningful substitutes have been found (Conrad, 2021). Recently, the WANT model of physical activity and sedentarism posited that the desire for movement behaviors is attributed to a combination of attempts to reduce tension (e.g., from drive and stress) and maximize pleasure and enjoyment (i.e., hedonic motivation; Seward, 1956; Stults-Kolehmainen et al., 2020a, 2022a). This idea deserves further delineation, but my basic postulate is that humans have a primary drive to move.

### Drive and drive theory

Drive theory is an early theory of motivation. The idea of drive is a “frontier” concept that straddles the physiology and psychology literatures (Conrad, 2021) and varies widely by field of inquiry (Katsafanas, 2018). It is traced back to the work of German physician Johann Friedrich Blumenbach (1752–1840) who used the term *trieb*, meaning force or impulse (Conrad, 2021). Notably, for this review, that term derives from an older word, *trieben*, meaning “to herd animals.” Nonetheless, the term is frequently associated with the works of Nietzsche and Freud (1964), who were primarily concerned with the drives of sex and aggression. Since this time, however, the concept of drive has been applied more expansively - making a concise definition difficult to find (Bridges, 1936). Some notable definitions follow here:

1. Clark Hull, who is most closely associated with Drive Theory, described drive as “motivation that arises due to a psychological or physiological need” (Hull, 1952).
2. Baumeister and Vohs (2007) describe it as “increased arousal and internal motivation to reach a particular goal.”
3. The American Psychological Association (2022) defines it essentially as a motivation state (Stults-Kolehmainen et al., 2020a), saying drive is “a generalized state of readiness precipitating or motivating an activity or course of action.” They add, “Drive is … usually created by deprivation of a needed substance (e.g., food), the presence of negative stimuli (e.g., pain, cold), or the occurrence of negative events.”
4. Seward and Seward (1937) defined drive as “an activity of the total organism resulting from a persistent disequilibrium.”
5. Seward described drive as an “excitatory state produced by a homeostatic disturbance” (Seward, 1956).
6. Conrad (2021) notes that “A drive is fundamentally a force, a pressure impelling the organism endogenously.” Speaking from a psychoanalytic viewpoint, Conrad also describes drive as, “bedrock psychical forces that attach to ideational content and are realized in dispositional states that induce affective, and so evaluative, orientations.”
7. Other perspectives come from: (a) Carver and White (1994) in their studies of behavioral activation, which consider drive as more similar to a trait, and label it as “persistent pursuit of desired goals,” (b) Lewin considered drive within his notion of “tension systems” (Marrow, 1977).

Consequently, the idea of drive is multifarious and difficult to operationalize. The most common elements from the definitions above are motivation, psychological states, goals, and homeostasis. A similar problem exists with the concept of stress, which conflates the ideas of stressors (i.e., impinging forces) with strain (i.e., the reaction to stress; Bartholomew et al., 2008; Lutz et al., 2010; Stults-Kolehmainen and Bartholomew, 2012; Stults-Kolehmainen et al., 2014, 2016; Stults-Kolehmainen and Sinha, 2014). In this case, drive has been blended, sometimes indiscriminately, with the ideas of: (a) needs, (b) deprivation of the need (i.e., a stressor), (c) felt tension and affective strain (i.e., a response), (d) a process of motivation, and (e) a motivation state. Given that the difficulties of stress have been met head on, I suggest that the concept of drive may also be pursued more rigorously. Here I define drive as, “a *motivation state* triggered by deprivation of a need or relevant environmental factors and associated with a subjective feeling of tension, functioning to help the organism maintain homeostasis.” [Note that, below, drive is often referred to as (a) a tendency to experience motivation states, which energize the organism to action through processes of deprivation, tension, consumption, and satiation or (b) as the *totality of these processes*.]

Given this lack of clarity, I find it best to start with the concept of needs and, specifically, primary needs. For Hull (1952), a need is a *biological requirement of the organism*, though Taormina and Gao (2013) more broadly assert that it is “characterized by, and defined as, a lack of something that is essential to an organism’s (a person’s) existence or well-being.” These researchers, who follow the paradigm from Maslow (1943, 1987), specifically note that “physiological needs can be operationally defined as the lack of chemicals, nutrients, or internal (e.g., exercise/health) or environmental (e.g., temperatures) conditions necessary for the body to survive, such that the extended absence of these things could lead to psychological stress or physical death” (Taormina and Gao, 2013). The generally accepted primary needs are food, water, oxygen, warmth, shelter, sleep, and sex (Taormina and Gao, 2013). Seward and Seward (1937) also include: activity, exploration (Butler, 1953), attack and self-assertion, escape, and submission. Clearly, there is no universal agreement on the numeration of the primary drives, and later Maslow (1987) urged ending the deliberation over the matter.

Needs are frequently categorized by the type of stimuli that are needed (e.g., water, food, movement). To help avoid circularity, “need” might be viewed as the lack of the necessary stimulus (Taormina and Gao, 2013). Lack of a necessity results in a state of *deprivation* or deficiency – a threat to homeostasis, and thus a stressor. There is an internal stimulus, a signal, as well as arousal associated with the deprivation. For instance, the condition of hunger is a motivation state produced by the deprivation of needed energy. It stimulates the sensation of being hungry, which is a signal to acquire food. Likewise, thirst is a motivation state, a condition, resulting from deficient water intake, associated with the feeling of being thirsty (see Table 1; Allen et al., 2017). Sensations produced by drive, like being hungry and thirsty, are almost always unpleasant and are considered sources of tension. Drive, however, is not simply the experience of tension. In Hull’s system, drive is the energy or motivational force that powers behavior (Hull, 1943, 1952). For Lewin (Marrow, 1977), drive was characterized as a “tension system.” It is an uncomfortable motivation state and resulting arousal, both generalized and specific to the need, with the objective to eradicate the deficiency in the need and focusing the attention of the deprived individual on a target. To acquire and consume the needed substance is to sate or satiate the deprived need, which ensures survival. The process is highly automatic and, theoretically, fundamental across all humans, perhaps all mammals, similar to core affect (Barrett et al., 2007).

**Table 1:** Some classic, primary (i.e., “genuine”) drives, including bodily movement and physical activity.

	Need	Behavior / action (voluntary)	Associated bodily functions (many involuntary)	Need state of deprivation (negative tension)	Descriptor	Physical manifestations and symptoms^*^	Psychological desire (positive tension)	Satiation state / descriptor
1	Food	Ingestion, eating	Digestion, absorption, defecation	Hunger	Hungry, peckish	Stomach growls and tightens	Appetite	Fullness, full
2	Water	Ingestion, drinking	Hydration, urination	Thirst	Thirsty, parched	Dry mouth and throat, headache	Water appetite	Quenched, slaked
3	Sleep	Sleeping	Rest, convalescence	Somnolence, fatigue	Sleepy, tired	Drooping, slouching	Sleep appetence	Rested
4	Sex	Copulation	Sexual arousal/receptivity, erection, orgasm	Sexual frustration	Concupiscent, aroused	Genital arousal, pacing and distracted movements, nocturnal emissions	Libido, arousal	Satisfied
5	Movement	Physical activity (PA), ambulation, exercise, moving	Non-exercise activity thermogenesis (NEAT); spontaneous PA	Restlessness	Restless, antsy, fidgety	Shaking muscles, stretching limbs, sweating, pacing, fidgeting, tapping	Appetence, activation	Exertiated ^†^

Exertiated is a stopping state, whereas “exerting” would be a starting or continuing state. “Exertiate” is the verb. In creating this term, we also considered “acti-fied,” the portmanteau of “active” and “satisfied,” but this may be confused with the word “actify,” which sometimes means “to start, activate or put into action”.

† Portmanteau of “exert”/“exercise” and “satiated”.

* There are generalized physical symptoms common to many/most needs (e.g., restlessness) as well as specific and localized ones.

### Criteria for primary drives

Specific criteria indicating what constitutes a primary drive are difficult to find, with the exception of Bridges (1936), which predates the classic work from Hull (1943, 1952). Based on considerations above, I propose that criteria would include:

1. Universality: The need is innate and common to nearly all humans, and possibly all vertebrates, similar to core affect (Hull, 1952; Spence, 1956; Barrett et al., 2007).
2. Biological requirement for survival: The need is necessary and not acquired or conditioned.
3. Emerges in deprivation: The drive is highly repeatable in response to the recurrence of need deprivation (Allen et al., 2017).
4. Subjective feeling of tension: Deprivation of the need has a psychological consequence. The condition of deprivation is typically unpleasant, like discomfort, which may be strong (e.g., an urge or craving) and may even be the dominant sensation in some situations. The tension is both generalized and specific to the need (e.g., stomach pangs in response to hunger).
5. Physical manifestations and symptoms: There are observable changes in the physical state, such as altered locomotor activity (e.g., the muscles shake in response to being antsy), along with emotional expression (e.g., being flushed).
6. Consumption: Behaviors are observed to consume the need (Allen et al., 2017).
7. Relief: Tension (thus, motivation) rapidly decreases with the provision of the needed stimulus (i.e., a “rapid diminution of motivational stimulus”; Seward, 1956).
8. Satiation and Over-consumption: A sense of being “full,” a motivational null-point, while having too much results in unpleasant sensations (Allen et al., 2017).
9. Developmental time course across the lifespan: The drive is exhibited early in infancy (or even before birth), is visible throughout infancy and childhood, and changes with aging (Bridges, 1936).
10. Under homeostatic control: The need has a circadian rhythm, typically. It is reproduced daily and may change seasonally (Budnick et al., 2022).
11. Responsiveness to other internal and external stimuli: Qualities of the internal environment (e.g., the body) and the external environment (e.g., either provocative or dull situations) can stimulate it (Loewenstein, 1996; Seli et al., 2014).
12. Approach and avoidance: Include aversions and appetites (e.g., humans crave fresh and avoid salty water; are repulsed by rotten food; Seward and Seward, 1937; Skinner et al., 2009; Stults-Kolehmainen et al., 2020a). Qualities of the stimulus matter as they are relevant for survival.
13. Biological mechanisms: There needs to be evidence of multi-system biological control to constitute a drive, including hormones, neuropeptides, brain circuitry, the microbiome, etc. (Allen et al., 2017; Dohnalová et al., 2022).

Drives differ from reflexes, instincts, basic psychological needs, impulses, habits, and predispositions (Seward and Seward, 1937; though in a circular definition, Szondi described drive as “an instinctual need that has the power of driving the behavior of an individual”; Szondi, 1972). Seward and Seward (1937) explain how drives differ from reflexes, “which are readily performed, such as blinking, coughing, and withdrawing from easily avoided cutaneous stimuli.” While there is some obvious overlap, drives differ from basic psychological needs, such as the basic need to belong (Baumeister and Leary, 1995; Tomova et al., 2020), and competence and autonomy (Ryan and Deci, 2007). Instincts differ from drives in that they can be easily externally provoked and do not need any conscious awareness. Conrad (2021) defines instincts as, “innate and unlearned biological processes realized in fixed action patterns that are direct expressions of the twin goals of natural selection: survival and reproduction.” A common example is a mother’s instinct to protect her young in danger (Bridges, 1936). The stress reactions of fight and flight, now more accurately reconceptualized as “freeze, flight, fight, or fright,” may be more pertinent (Bracha et al., 2004). The idea of “impulse” is similar to drive in that it has been poorly defined, but ostensibly these are separate but potentially related constructs (Gardner, 2015; Brand and Ekkekakis, 2018; Conrad, 2021). For instance, both drive and impulse may be related to a third construct, urge, which is a feeling of strong desire to approach an object or behavior.

Importantly, drives may or may not have an object or a precise target. “There is no inherent correspondence between a drive and its object in the same way that there is between an instinct and its object” (Conrad, 2021). Conrad goes on to add, “drives are fundamentally forces and may be ‘continuously flowing’ without any attachment to an object.” On the other hand, Conrad also notes that drives may be vector-like, having both force and direction. In line with these notions, the drive for movement may be expressed in a multitude of ways, such as a longing for sport, needing to go for a walk or wanting to get fit and more muscular (i.e., all having direction/ an object), or a *pressing readiness to fidget and move about* (i.e.*, not having direction / an object*).

### Movement as a primary drive: Criteria 1–3

#### Movement is needed for survival

My initial arguments that humans have a basic drive to move were published in a former paper (Stults-Kolehmainen et al., 2020a) that, while providing a strong rationale, fell short of emphasizing that movement might be characterized as a *primary drive*. To do so would be to imply that movement is necessary for survival – that there is a physical need – which is nearly universal (criteria 1 and 2, above). The study of drive has frequently been explored in rodent models, where there is strong evidence of a basic drive to move (Garland et al., 2011). However, in an early review on the matter, Lore (1968) has argued that there is inconsistent evidence of an innate drive to move in rodents. However, he included studies using activity deprivation ranging from 5 h to 100 days, thus conflating acute and chronic responses.

Thus far in humans, movement has atypically been included in lists of basic physical needs and thus, primary drives, such as eating, drink, sex, social interactions, etc. (Hull, 1952; Loewenstein, 1996; Tomova et al., 2020). Nevertheless, Bridges (1936), Seward and Seward (1937), and more recently Taormina and Gao (2013) have been direct in the assertion that physical activity (and exercise) is a primary need and drive. Others have been highly suggestive of the point (Tolman, 1932; Collier, 1970; Feige, 1976; Rowland, 1998, 1999, 2016; Reiss, 2004; Schultheiss and Wirth, 2008; de Geus and de Moor, 2011; Kalupahana et al., 2011), and it has been partly established above. In short, movement is necessary for instrumental reasons, for play (Rowland, 1998; Rowland, 2016), exploration for acquisition of rewards, and experiencing and processing novel environmental stimuli (Butler, 1953; Panksepp, 2006; Cabanac, 2006a,b; Schultheiss and Wirth, 2008; Panksepp and Biven, 2012; Frijda, 2016; Parker et al., 2020). There is also a general need for stimulation (Ekkekakis, 2013), such that individuals will even subject themselves to painful electric shocks when faced with nothing else to occupy their minds (Wilson et al., 2014). Ekkekakis (2013) summarized older arguments that indicated that humans have an “inherent propensity,” “susceptibility” or “drive for activity.”

#### Clinical manifestations

A case for “drive for activity” has also been made in certain clinical conditions, such as anorexia nervosa (Davis and Woodside, 2002; Scheurink et al., 2010; Casper, 2018, 2022). Aversive sensations associated with a lack of movement are also hallmarks of various disorders, such as Restless Leg Syndrome (Garcia-Borreguero et al., 2011; Khan et al., 2017), akathisia (Iqbal et al., 2007), exercise addiction/dependence (Hausenblas and Downs, 2002; Ferreira et al., 2006; Garland et al., 2011; Lichtenstein and Jensen, 2016; Stults-Kolehmainen et al., 2022b), and hyperactivity (Willerman, 1973; Scheurink et al., 2010). Some of the earliest observations of this go back over 100 years, such as a case of a girl with akathisia who was rarely able to stop moving (James, 1907). With hypokinetic disorders, such as Parkinson’s and depression, there may be a total loss of desire and urge to move, characterized as motor apathy or psychomotor retardation (Sinha et al., 2013; Busch et al., 2016; Stults-Kolehmainen et al., 2022b). Collectively, I have called the disorders characterized as very high or very low in motor urge as Movement Urge Dysfunction Disorders (MUDD), which seem to fall along a movement urge dysfunction spectrum (MUDS; Stults-Kolehmainen et al., 2022b). This proposition is supported in the Parkinson’s literature and elsewhere as conditions characterized by hypo- and hyper-dopaminergic states in the cortico-striatal circuits (Sinha et al., 2013). Until recently, however, a strong case was not made for the general population of healthy adults (Stults-Kolehmainen et al., 2020a, 2021b, 2022a). To support the point, one would need to assert that movement is (has been) a common need for all (most) humans and is (has been) needed for survival – not requiring conditioning. Thus, the first two criteria seem to be satisfied.

#### Movement deprivation

It appears that the 3rd criterion from the above list (i.e., deprivation) is met as well. Seward and Seward (1937) note that with “prolonged rest, the refreshed organism requires activity to restore equilibrium.” Arousal emerges when movement is deprived, such as prolonged sitting and physical restraint (Zuckerman et al., 1968; Ravussin et al., 1986; Reardon et al., 2008; Bouwens et al., 2018), which is highly repeatable and has been observed in primates (Butler, 1953) and humans (Stults-Kolehmainen et al., 2021b, 2022a; Budnick et al., 2022). Under restrained conditions humans feel “intense uneasiness or craving” or “pressing readiness” or tension, perhaps similar to appetite (Loewenstein, 1996; Ferreira et al., 2006); a comparison propelled by Rowland (1998). Almost anyone can identify with the discomfort of sitting for prolonged periods, feelings of being antsy, jittery, squirmy, restless and/or fidgety, and the relief provided by movement (Levine et al., 2005; Seli et al., 2014). They are also observed in various conditions, such as forced bed rest (DeRoshia and Greenleaf, 1993; Ishizaki et al., 2002), loss of playtime/recess (Jarrett et al., 1998), being constrained during an MRI scan or metabolic testing (Ravussin et al., 1986; Aoyagi et al., 2003; Heilmaier et al., 2011), sudden decline in one’s usual exercise routine or mandated detraining (Mondin et al., 1996; Sugawara et al., 2001), quarantine / confinement (Zuckerman et al., 1968; Shalev, 2008; Blacutt et al., 2021; Stults-Kolehmainen et al., 2021a; Filgueiras and Stults-Kolehmainen, 2022), and sensory deprivation (Morgan et al., 2013).

Here are some specific examples:

1. The use of physical restraints in older adults, ostensibly to prevent falls while moving, causes large increases in anxiety and exacerbates agitation (Scherder et al., 2010).
2. Solitary confinement with movement restriction results in dramatic effects to physical and mental health, and, concordantly, international law mandates 1 h of exercise time per day (Shalev, 2008). As movement is a basic need, it is also considered by some to be a human right.
3. College-aged male participants who were physically restricted with straps to a form-fitting bed for 8 h had large increases in somatic complaints, feelings of discomfort, and “muscle tightness, sweating, pain in joints, urge to urinate, and itching” (Zuckerman et al., 1968).
4. Even in restricted environmental stimulus training (REST – a float tank designed for sensory deprivation), which is designed for relaxation, “a tension develops which can be called a ‘stimulus-action’ hunger; hidden methods of self-stimulation develop: twitching muscles, … stroking one finger with another, etc.” (Lilly, 2022).
5. In their studies of daily energy expenditure, Ravussin et al. (1986) and colleagues constrained participants to a nondescript metabolic chamber for 24 h and did not permit them any kind of physical exercise, which resulted in increased spontaneous physical activity (SPA). They noted that, “Because the subjects were not allowed to carry out physical exercise, such as isometric exercises or calisthenics, it is possible that such activity represents an unconscious need to be active.”

#### Activitystat – the physical activity set point

Consequently, there may be an energy expenditure set point, under biological control, resulting in drive to move when the level of required activity is not met, and lower drive when there is too much or sudden bursts of activity (Rowland, 1998, 2016; Garland et al., 2011). Bennett (1995) wrote of the general idea of biological control of movement,

“As with breathing, elimination, and sexual activity, there can be considerable ambiguity about the degree of volition in the timing, frequency, and circumstances of any particular act of eating or exercise. In the moment, snacking [and movement] may appear to be altogether subject to conscious control; in the aggregate, however, such behavior assumes a certain biologic inevitability.”

The drive for movement may, alternatively, be a mere consequence of the drive for energy homeostasis, or central nervous system stimulation (Rowland, 1998; Wilson et al., 2014). Rowland coined the term “activitystat” in reference to a homeostatic center of control that keeps total daily energy expenditure (TDEE) relatively stable. To maintain the set point, subtle and perhaps unconscious adjustments may be automatically applied to modulate resting metabolic rate, incidental or spontaneous physical activity, as well as voluntary exercise. There is mixed evidence that when exercise suddenly increases, NEAT (i.e., kcals of non-exercise activity) may decrease (King et al., 2007; Westerterp, 2018). A recent study purporting to test the activitystat model with various training protocols did not find support for the idea (Gomersall et al., 2016). However, a test of this model with provision of activity, but not deprivation of activity, is a poor test of the framework. There is less direct evidence that sudden declines in activity may result in increases in caloric expenditure, but it is possible that this is due to insufficient methods of investigation.

### The subjective feeling of drive (felt tension): Criterion 4

Drives are associated with strong signals and subjective feelings of urge and tension, which change rapidly with consumption, satiation, and over-consumption. If humans have a drive to move, what is the proper term for the feeling of tension associated with its deprivation? A lack of food results in hunger and feeling hungry; a lack of water, thirst and being thirsty, a lack of sleep, fatigue and feeling tired (see Table 1). Historically, a lack of movement was not a problem, and thus no strong word is associated with the state of deprivation, at least in English. Older literature has utilized terms, such as “necessity of body exercise,” “volitional promptings,” “intense uneasiness,” “craving,” and “pressing readiness” (Bain, 1855; Baldwin, 1891, 1894; Shirley, 1929; Hill, 1956; Finger and Mook, 1971). Several candidates might be worthy to succinctly describe “feeling deprived of movement”: feeling antsy, fidgety, driven, cooped up, hyper, wired, agitated, on edge or edgy, restless, squirmy, jittery, wound up, keyed up, stir crazy, having cabin fever, or other overlapping terms also referring to being energized (Jarrett et al., 1998; Emerson, 2020; Stults-Kolehmainen et al., 2022b). Just like psychosomatic sensations of fatigue and energy, feelings of restlessness could be construed as being solely physical and/or mental (Herring and O'Connor, 2009). Sensations of energy and fatigue are moderately correlated with desires and wants to move and rest (Stults-Kolehmainen et al., 2021b). Feeling antsy has connotations of an external source of stimulation (i.e., literally “ants in the pants”), and “cooped up” typically refers to conditions of constraint, while being “wired” usually refers to consumption of excess caffeine or other external stimuli (Levitt et al., 1993) and “feeling hyper” often describes responses to medications (Hauser and Zesiewicz, 1997; Rabkin et al., 2004). Many other terms also exist: drive for activity (Casper, 2018, 2022), urges, cravings, and appetence (Ferreira et al., 2006). The collection of these has been generally referred to as “affectively-charged motivation states” (ACMS) for physical activity (Stults-Kolehmainen et al., 2020a, 2022a). Lastly, it’s important to note that for those with exercise addiction/dependence, abstaining from movement may result in sensations of withdrawal and cravings for exercise (Hausenblas and Downs, 2002; Garland et al., 2011; Lichtenstein and Jensen, 2016). Overall, “urges to move are well-documented in situations where such sensations are bothersome and unproductive” (Stults-Kolehmainen et al., 2022b).

### Criteria 5–12

For criteria 5–12, I provide brief evidence.

Criterion 5 (Physical manifestations): Studies of participants in situations of deprivation, mentioned above, discuss various physical responses, like spontaneous and nervous fidgeting (Ravussin et al., 1986). In interviews, undergraduate honors students have indicated that they feel mental and physical symptoms, such as being jittery and antsy, with legs stretching out and twitching when deprived of movement (Stults-Kolehmainen et al., 2022a).

Criterion 6 (Consumption): Empirical evidence specifically connecting the constructs of need, deprivation, felt tension, and resulting behavior is lacking. However, motivation states to move (i.e., desire, want, urge, craving), as measured by the CRAVE scale (Stults-Kolehmainen et al., 2021b), were associated with intentions to be active in the following 0–30 and 30–60 min time frames (Budnick et al., 2022).

Criterion 7 (Relief): With consumption (e.g., moving), there should be reductions in negative affect built up from deprivation. As one example, older adults who were released from restraints and allowed to move and exercise experienced reductions in agitation and arousal (Scherder et al., 2010).

Criterion 8 (Satiation and over-consumption): Excessive movement is associated with fatigue, soreness, pain, and alterations to locomotion, which may be construed as signals of satiation with movement (Stults-Kolehmainen and Bartholomew, 2012; Stults-Kolehmainen et al., 2014, 2016), but are not necessary for satiation to manifest. Satiation might be better indicated by alterations to motivation states. With a maximal treadmill test, desires to move dropped 24%, and desires to rest increased 74%, both of which were large effect sizes (Stults-Kolehmainen et al., 2021b). Evidence of satiation also comes from compensation studies, which show that when activity is very high, NEAT decreases (Garland et al., 2011; Westerterp, 2018). The formally constructed notion of exercise satiation is a recent development that has been typically applied to eating disorders (Barker et al., 2022) but likely also applies for healthy populations. My colleagues and I have coined the term “exertiated” to denote when a person feels satisfied and/or satiated with physical activity (see Table 1).

Criterion 9 (Lifespan development): Movement tendencies are observed even in the fetal stage and are considered predictors of infant health (Perry et al., 2022). Movement, muscle tone, and reflexes immediately after birth are likewise important. According to Bridges (1936), the drive to move rhythmically is exhibited in the first month of life, “General exercise and rest are the first noticeable forms of infant behavior,” the purpose being, “sensory exploration and utilization of the environment.” She notes that the “drive for locomotion” (ambulation) is exhibited at 12–24 months, and these drives are related to the need for more advanced exploration and exploitation of the environment. Rowland has commented extensively on drive for movement in children that wanes with adolescence (Rowland, 1998, 1999, 2016).

Criterion 10 (Homeostatic control): Movement varies systematically over the course of the day, week, and year (Beighle et al., 2008; Budnick et al., 2022). Recent data from adults monitored 6 times a day for 8 days indicates that motivation states to move are like a biorhythm for over 80% of people. In other words, the majority of individuals have a circadian curve for movement drive (Budnick et al., 2022), similar to eating and sleeping. See above for the related idea of “activitystat.”

Criterion 11 (Responsiveness to internal and external stimuli): Exogenous factors, such as daylight, caffeine, illicit drugs, prescription medications, and music, highly influence locomotion (Levitt et al., 1993; Hauser and Zesiewicz, 1997; Kaplan et al., 1997; Rabkin et al., 2004; Tucker and Gilliland, 2007; Stults-Kolehmainen et al., 2020a, 2022b). Internal stimuli, like joint pain in individuals with low back pain disorders, regularly propels them to fidget and shift their bodies, sometimes multiple times every minute, in order to relieve pressure and avoid discomfort (Dunk and Callaghan, 2010; Beitel et al., 2016). Psychological stress results in displacement behaviors, such as pacing and stroking one’s hair (Troisi, 2002; Mohiyeddini et al., 2013). A lack of stimulation or monotony may also result in activation of drive (Seli et al., 2014).

Criterion 12 (Approach and avoidance): Humans have an innate desire to move, particularly in youth, but they also have strong aversions for movement. For instance, when in the thralls of physical pain (e.g., in this case, before movement even starts) most people actively avoid movement – the extreme of which is kinesiophobia (Lundberg et al., 2006; Beitel et al., 2016; Glaviano et al., 2019; Stults-Kolehmainen et al., 2020a). Also, physical labor and exercise in vigorous intensities are sources of punishing sensations, which are in themselves drive to stop moving. However, physical work and its sensations can be conditioned to have less impact. Brown (1955) attempted to explain the interactions between drive (in this case, drive to stop moving and rest) and conditioning (e.g., to move more).

“Under some conditions, it might be predicted that the intense [and painful] proprioceptive stimulation and [fatiguing] muscular strain due to prolonged work should have drive-like effects [to stop and/or avoid movement]. But if an organism gets appropriately reinforced training, it can acquire a tolerance for the stimulative effects of repetitive muscular effort that is little short of astounding. Rats and pigeons can be trained to make hundreds of responses for a single bite of food if the percentage of reinforcement is high initially, and if the reduction in frequency of reinforcement with further trials is sufficiently gradual. In such instances, apparently, the stimulation accruing from a multitude of successive reactions does not function as a drive [to stop movement], since behavior, such as resting, though followed by the cessation of such [painful] stimulation, is not strengthened.”

Robinson and Berridge (2013) have demonstrated how aversive and punishing sensations can be transformed quickly into strongly desired stimuli *via* the activation of mesocorticolimbic circuitry, which ostensibly applies to physical activity as well.

### Movement as a secondary drive

Primary needs and drives are distinguished from secondary needs and drives, the latter of which are not directly needed for survival but help to optimize survival. Secondary needs have also been referred to as “quasi-needs” (Marrow, 1977). Hull asserted that some reinforcing behaviors, like seeking money, are secondary drives (Hull, 1952). According to Baumeister and Vohs (2007), “Secondary or acquired drives are those that are culturally determined or learned, such as the drive to obtain money, intimacy, or social approval.” Bridges (1936) adds, “Any acquired habit is a drive to some extent,” which is influenced by development; “The primary drives become further differentiated and directed towards varying specific ends with increasing age.” It seems likely that some movement behaviors (e.g., structured exercise) fall within this category. It should be noted that in previous manuscripts, I have indicated that wants and desires for movement may be primary (i.e., want for movement itself) versus secondary (e.g., want to move in order to achieve something else; Stults-Kolehmainen et al., 2022b). For instance, one may feel antsy and want to move (i.e., a primary want or desire), or one may want a drink of water and thus feel urged to get up and go the kitchen (i.e., a secondary want or desire).

### The influence of drive on behavior

How does drive influence behavior? Nietzsche provided one way of describing how drives impact action, which is concordant with the notion of incentive salience (Berridge and Robinson, 1998; Salamone and Correa, 2002). As described by Conrad (2021),

“The drives (1) identify features of the world as salient, (2) induce an affective response to that object [e.g., an urge or craving] that (3) justify a certain evaluation of the world consonant with that affective response, and (4) impel one toward a certain (set of) behavior(s). Put more parsimoniously, the drives are dispositional states that induce evaluative orientations.”

#### Hull’s formula

Hull (1943, 1952) was interested in creating precise formulas to predict behavior from drive, predicated on the idea of stimulus–response. In this case, the response was the “excitation potential” (_S_E_R_) - the likelihood that stimulus (s) would result in response (r). His basic formula included: (1) drive strength (D), which was essentially the time of deprivation, and (2) habit strength (_S_H_R_), conditioned from repeated reinforcing trials (Martens, 1971). Later, other factors in his formulas included: (3) intensity of the stimulus triggering the behavior (V), such as light or a bolus of carbohydrate, and (4) incentive (the potential pleasure that the stimulus can provide; K). Further developing more complex models, Hull added: (5) any delay to acquire the stimulus, (6) reactive inhibition or satiation resulting from continued exposure to the stimulus, (7) conditioned inhibition that does not dissipate over time, and other factors, such as the “reaction threshold.” Importantly, the strongest responses are for stimuli that reduce tension (i.e.*, negative reinforcement*) *while also enhancing pleasure* (i.e., *positive reinforcement*; Seward, 1956; Allen et al., 2017).

How this might apply to movement could be demonstrated in the following example. A child constrained to a desk will be conditioned to sitting (_S_H_R_) as part of adapting to schoolwork but will also freely engage in movement during recess. A lack of movement over the course of morning studies results in drive (D) – a motivation state. Approaching the hour of recess is a stimulus that will activate an anticipatory response (V), and the incentive of playing a fun game provides a powerful forecasted reward (K). Moreover, any delay in recess beyond the normal time will result in growing arousal and discomfort (i.e., tension). “Drive” here may be observed with physical manifestations of tension, such as fidgeting, pacing, swaying, shifting of the body, sweating, etc. This affect may be modulated, however, by additional training to sit still. The eventual engagement in play will, more than likely, swiftly result in reduced tension, as predicted by Hull (1952).

#### Drive reduction theory

Hull’s drive theory was not just a theory of how the organism responds to deprivation of needs (Hull, 1952). His Drive Reduction Theory was a paradigm of motivation, learning, and the development of habits. The organism is motivated to eliminate aversive feelings, which he later called cravings, and will behave to do so (Hilgard and Bower, 1975). In the case of hunger, it will feel peckish and will search for food (*via* movement) to reduce those pangs. Inevitably, the deprivation occurs again, and the organism repeats the behavior that previously reduced the drive, developing a habit. Importantly, reducing a sensation of drive was the principal reinforcement for human behavior (and not merely the attainment of pleasure, for instance).

Several criticisms of Drive Reduction Theory led to its relegation.

1. It is highly formulaic and unwieldy, and experimentation from Skinner provided cleaner experimental design (Skinner and Morse, 1958; Smith, 2000).
2. Not all human behavior has the goal of reducing tension. Clearly, humans are highly driven to attain pleasure (Young, 1966) and not just reduce arousal.
3. On the other hand, it is easy to observe contra-hedonic processes, in other words, pain seeking (Riediger et al., 2009).
4. Drive Reduction Theory was principally sidelined, however, because of observations that consumption of wanted and needed stimuli did not always result in reductions in drive and arousal.

To follow this last point, sometimes, there is increased arousal with consumption. A famous example is a person who leaves the comfort of sitting to ride a roller coaster – the sensation seeker looking for more tension (i.e., arousal) – not less (Brown, 1955). In the school recess example above, Hull’s theory, as modified by Brown (1955), predicts that the play will result in increased arousal. Since this time, sensation seeking has been well accepted (Babbitt et al., 1990a,b; Rhodes and Smith, 2006). More recently, the idea that “some like it vigorous” (Ekkekakis et al., 2005); in other words, some people prefer strenuous levels of activity and can readily tolerate high intensities. Importantly, it appears that Hull conflated the concepts of displeasure with arousal in his ideas about tension. In these examples, there will be strong reduction in negative affect (i.e., reduced sense of displeasure) and enhanced positive affect, as commonly observed in studies of exercise (Jones and Zenko, 2021). Consequently, Drive Reduction Theory has faded; nevertheless, the idea of drive has persisted because it aligns with observed data (Allen et al., 2017).

### Affectively-charged motivation states and the WANT model

Can Drive Reduction Theory be rehabilitated and melded with the widely accepted Hedonic Theory? In his thorough critique of both frameworks, Seward (1956) acknowledged that, “Since the two theories are not strictly incompatible, it is possible to accept both. Indeed, the foregoing evidence strongly indicates that both drive reduction and incentive play a part in reinforcement.” Recent work has attempted to combine classic work on aspects of drive theory with hedonic theory and other recent frameworks of physical activity behavior (Brand and Ekkekakis, 2018; Stults-Kolehmainen et al., 2020a). I highlight the Wants and Aversions for Neuromuscular Tasks (WANT) model, which has been discussed extensively elsewhere (Stults-Kolehmainen et al., 2020a,b, 2021b, 2022a,b; Budnick et al., 2022; Filgueiras et al., 2022). In the WANT model, humans are motivated to reduce tension (as in Drive Reduction Theory) and to approach pleasure (as in Hedonic Theory; Young, 1966). Moreover, this model emphasizes the subjective feelings of wanting or desiring to move, which are called affectively-charged motivation states (ACMS). ACMS to move may be felt weakly, such as a want, or strongly, such as an urge or craving. Importantly though, while drive sensations (e.g., tense arousal), which are considered motivation states, are typically considered unpleasant, sensations of wanting to move may be experienced as pleasant. This is well documented, for instance, in the concepts of groove and swing (Janata et al., 2012, 2018; Stults-Kolehmainen et al., 2022b), which are the ability of music to stimulate pleasurable desires to move the body. Thus, ACMS and the WANT model incorporate affective tone. Interestingly, in a recent study, arousal was a stronger predictor of motivation states to move than affective valence (Budnick et al., 2022), and arousal and affective valence did not interact to predict ACMS (likely because the study was underpowered). Lastly, the WANT model includes dimensions of (A) Move versus Rest motivation combined with (B) approach and avoidance motivation (Skinner et al., 2009). In other words, there is also a drive to “not move” (see Criterion 12). Addressing both, Bridges (1936) comments, “[The child has] a tendency to arrestation of movement upon sudden extensive change or intense sensory impact. Accumulated experience makes of this reaction a drive to avoid the obnoxious and whatever threatens personal security,” and “Advantageous rest pauses between explorations.”

### Problems with the concept of drive for movement

There are several serious challenges to the idea that bodily movement and exercise are basic needs resulting in drive processes. These have been addressed in other papers (Stults-Kolehmainen et al., 2020a, 2021b) but are worth extending in this current manuscript. To start, one might believe that movement is simply a *bodily function*, just like breathing, urination, or defecation – something organisms do that is devoid of motivation. However, one might make the case then that sexual behavior is simply a bodily function. Nonetheless, it is typically included as a drive - as it is essential for survival, at least as a species, but also because later in life progeny assist in extended care. In some sense, movement may be an even higher drive, a *superordinate drive* because it is necessary to acquire food, water and to engage in sex, etc. (the same case could be made for breathing). A second issue is that the drive to move is conflated with psychological drives, such as (a) the need for autonomy and independence (e.g., children learning to stand, walk), (b) as well as the needs for competence and productivity (e.g., attempts to accomplish things), (c) the need for social interaction (Tomova et al., 2020), and/or (d) the need for stimulation and sensation seeking (e.g., a need for thrills; Babbitt et al., 1990a,b; Watten, 1997; Rhodes and Smith, 2006; Ryan and Deci, 2007; Wilson et al., 2014). For example, conditions of being “cooped up” and “stir crazy” likely combine noticeable increases in the drive to move along with concomitant upticks in the drive for social interaction (Tomova et al., 2020). It is not within the scope of this manuscript to address this issue, but it could be the case that drives and wants are hierarchical, similar to the hierarchies delineated by Maslow (1943, 1987), Taormina and Gao (2013), or Sonstroem and Morgan (1989).

#### Does a drive to move need to exist?

A more significant critique could be stated as, “movement is just the means to the ends.” In other words, movement is not reinforced in itself, and people do not move just for the sake of moving. To put it differently, movement is merely instrumental or utilitarian. It is true that movement accomplishes many things for us– it is a means to an end. It accomplishes, up until the modern age, almost everything needed for sustenance. Through movement, we may acquire food, labor to produce goods, propagate, even shake our muscles to become warm (Garland et al., 2011). Moreover, movement allows us to not only exploit, but also explore our world (Butler, 1953; Inzlicht et al., 2014) and to remove obstructions in the process. As Bridges (1936) notes, humans have a “strong utilitarian drive for adaptive exploitation” – accomplished by our limbs. Consequently, one might conclude that it requires no reinforcement as well as no drive to energize it.

#### Would a [strong] drive to move do more harm than good?

As established above, movement is very useful, but it comes at a cost and must be counter-balanced. Humans also strive to “avoid undue expenditure of energy,” and “rest allows other interests to come into force and direct behavior” to allow “processes of organization and action planning or thought” to take place (Bridges, 1936). If movement was as lucrative as food or sleep, we may not stop, which would detract from other needs, mitigate adaptation and possibly be destructive to the body. Indeed, this is substantial risk for those with akathisia or exercise addiction/dependence (Lichtenstein and Jensen, 2016), and most well-trained athletes know the importance of balancing exercise and rest. Consequently, while classic drives have strong signals, like the drive for food (pangs or hunger) or the drive for sleep (tiredness), humans have developed relatively weak signals to move (as well as relatively weak signals to *not* sleep - e.g., when awakening). Furthermore, movement has been so instrumental and necessary for survival that strong signals to move were unnecessary. Up until the modern time, the drives for food, water, etc. (with their signals) were mostly sufficient to initiate and maintain necessary movement. Rather, we have strong signals to stop movement (i.e., pain and soreness). Nevertheless, the urge to rest is not simply a contra-drive in opposition to movement, but rather works in concert with it to maximize adaptation, an idea gaining greater ground (Stults-Kolehmainen et al., 2020a, 2021b, 2022a).

#### Appetite and appetence for movement

All of this being said, how do we rectify these issues with the fact that some people, and maybe many people, do move just for the sake of moving? Even when it is not necessary to move, people do it anyway. Likewise, people eat, rest, and have sex, even when there is no compelling need to do so, and there is no buildup of tension to release. These activities may all provide a source of pleasure, and engaging in them is not to just rid oneself of tension. However, it is generally agreed that movement and exercise are not highly pleasurable for most people, and aversions associated with movement may certainly be a large barrier for movement for some (McBeth et al., 2010). On the other hand, movement may not be a large source of aversive sensations either. With the balance of reinforcement at play, it might be concluded that movement is generally agreeable (Garland et al., 2011; Stults-Kolehmainen et al., 2020a). One can imagine that people have an “appetite” to move (Rowland, 1998), which has been called “appetence” (Ferreira et al., 2006). The drive and desire to move does exist, mostly because bodily movement is the vehicle by which we accomplish things, but it is regulated relatively without notice, usually only rising into awareness when it becomes disordered (e.g., Restless Legs Syndrome) or bothersome (with prolonged deprivation; Stults-Kolehmainen et al., 2022b) or when a person is specifically queried about it (Stults-Kolehmainen et al., 2020b, 2021b, 2022a).

#### If humans have a basic drive to move, why do not they?

This is a particularly vexing issue for some of those who are least active – middle-aged to older adults from WEIRD (i.e., Western, educated, industrialized, rich and democratic) populations (Henrich et al., 2010). One might argue that the need to move is a phenotype heterogeneously distributed across the population. I believe that a stronger argument, however, is again from Bridges (1936), who argues that drives are developmental and vary across the lifespan. It is obvious that most children have a strong drive to move. Easy is it to find a child that enjoys frolicking – engaging in playful movement, which is highly stimulating for growth, maturation and socialization (Stults-Kolehmainen et al., 2020a). Movement seems to be a strong drive from birth, peaking around the age of maximal physical development. In this regard, as one ages, the urge to move declines as it is less useful to develop the body and mind – there is a switch from an emphasis on development to maintenance and slowing down decay. In short, movement loses its “adaptive value” as one ages. Middle-aged and older people ostensibly experience diminished drive to move, and there is some evidence from a study that followed people for 2 years that the desire to move decreased with age (Stults-Kolehmainen et al., 2021b). It may also be the case that drive is attenuated through repeated pressure in childhood to remain still and control impulses (Seli et al., 2014). Observations such as these do not lead to a conclusion that there is no drive to move. Consider another basic drive – sex. Like movement - desire for sex varies across the lifetime. Children have apparently no drive for sex, and it ostensibly diminishes with age for most of the population. Simply put, just because children, the infirm and elderly have minimal libido does not mean that sex drive does not exist.

Drives are malleable to time and place - and certainly era as well. As with previous comments above, one should note that rapid technological advancement has changed the dynamics of desire, but not expunged them. As Bridges states (Bridges, 1936), a drive “undergoes processes of development and change of form in response to environmental condition.”

Changes may follow several routes. Drives can be counter-conditioned according to Hull’s (1943, 1952) basic formulas. For instance, children usually learn to sit and be still. Drives are also highly responsive to internal and external (i.e., endogenous and exogenous) environmental stimuli (Loewenstein, 1996). The modern, obesogenic world provides an overload of stimulation, but perhaps a lack of natural environmental stimuli (e.g., light, to which movement was ostensibly paired; Lake and Townshend, 2006; Wilson et al., 2014). Competing stimuli and drive may overpower (i.e., overtake) or overshadow (e.g., drown out) drive for movement. Alternatively, the need for movement may be satiated by digital movement stimuli provided in rich content from social media memes, short video clips, television, and video games (Rowland, 1998). The average person is also much larger than before (Wong et al., 2022), and this increased size is associated with aversions to movement, such as painful sensations in the joints, skin friction, and other nuisances (Speck et al., 2014; Purim et al., 2015).

### Future research

#### Negative reinforcement

Rapid progress is being made in theory development for physical activity, exercise and sedentary behaviors. However, the dominant perspective at this juncture is on affect and the reinforcing power of pleasure (Ekkekakis and Zenko, 2016; Williams and Bohlen, 2019; Jones and Zenko, 2021). Emphasis is also placed on punishment (experience of pain) and aversions associated with exercise, typically above ventilatory threshold (Ekkekakis et al., 2005, 2011). Negative reinforcement is typically considered in terms of reductions in anxiety and depressed mood and the analgesic effect of exercise (for some people in some situations; Bartholomew et al., 1996, 2005; Busch et al., 2016). Rarely are the various sources of displeasure comprehensively included, however (Backhouse et al., 2007). In short, most models of physical activity motivation do not include basic drive, which may be a source of considerable displeasure, arousal, and tension for many people, particularly those who are healthy and younger, but also for those suffering from a wide range of conditions (Stults-Kolehmainen et al., 2022b). Future developments should consider all of these factors (see Table 2).

**Table 2:** Examples of sensations of tension that may result in physical movement.

		Source of stimulus^*^	
		Internal (Innate/Endogenous)	External (Exogenous)
Valence of stimulus	Negative	Drive, pain, depressed mood	Work stress, social anxiety
	Positive	Runner’s high	Groove^†^

* See Seward and Seward (1937).

† Sensation of wanting to move in response to music.

#### Appetitive versus reflective desires

Future research should consider interactions between: (1) appetitive (e.g., hedonic-oriented) and reflective (e.g., cognitive) sources of desire (Williams and Bohlen, 2019), (2) need states versus appetence, (3) primary versus secondary drives/wants and, (4) the various primary drives. For instance, when satiated with food, individuals report lower desire to move (Budnick et al., 2022), which may be related to processes of digestion. Basic drive, however, is not dependent on appetite or reflection and is highly automatic; thus, it should be considered within the domain of automaticity and Type 1 behavioral processes (Brand and Ekkekakis, 2018). Lastly, could subjective feelings of drive to move be misattributed to other sources of tension? Feelings of being antsy (due to a lack of movement) could be attributed to external sources of stress, such as work anxiety.

#### Measurement

Measuring drive continues to be a challenge and varies with one’s definition of it (see above). The behavioral manifestations (e.g., locomotion) may be measured by direct observation (Galton, 1885) or with subjective (Seli et al., 2014) and objective measures of fidgeting, such as Nintendo Wii Balance boards (Seli et al., 2014). Tension, the result of deprivation (i.e., of movement) may be detected indirectly through tools that have been developed to measure affective valence (e.g., Feeling Scale) and arousal (e.g., Felt Arousal Scale). The transient want or need of movement (i.e., the subjectively felt motivation state) may also be measured with the CRAVE and ARGE scales, which have 13-item and 2-item versions (Stults-Kolehmainen et al., 2021b; Filgueiras et al., 2022). There is no known instrument to measure associated feelings (e.g., fidgetiness, restlessness, antsiness) and motor changes, such as trembling, shaking, raking of the limbs, etc. (Tiidus, 2008). Direct observation in the laboratory is sorely needed to witness and document these phenomena.

#### Mechanisms

These data should be combined with biomarkers likely associated with drive for activity, including orexin (i.e., hypocretin), leptin, testosterone, cortisol, dopamine, activity in the nucleus accumbens, ventral tegmental area and substantia nigra, vagal drive/HRV/RSA, genetics and the microbiome (Willerman, 1973; España et al., 2002; Salamone and Correa, 2002; Aoyagi et al., 2003; Furlong et al., 2009; Scheurink et al., 2010; Garland et al., 2011; Casper, 2022; Dohnalová et al., 2022). There is likely huge inter-individual variability in drive, which may be related to stress coping style, temperament, impulse control, personality, and other factors (Rhodes and Smith, 2006; Garland et al., 2011; Seli et al., 2014). Drive for specific types of movement, such as moving in short spurts, or for extended periods of time (Belke and Garland, 2007), may be associated with properties of muscle and may be conditioned as well.

### Application

#### To research methods

The concept of drive might have general application for experimentation and data interpretation in exercise science. Frequently, study participants are asked to refrain from exercise before assessments and training protocols (Aoyagi et al., 2003; SantaBarbara et al., 2020) and/or to take time off from their normal routines (Stults-Kolehmainen and Bartholomew, 2012; Stults-Kolehmainen et al., 2014, 2016). This is ostensibly to ensure that participants have adequate energy, no muscle damage, and any effect of previous training is “washed out,” particularly in within-subjects, randomized cross-over trials (Ballmann et al., 2021). However, it should also be considered that depriving movement for individuals accustomed to physical activity results in increased drive or motivation state, perhaps with noticeable increases in arousal, desire to move, and alterations to locomotion (e.g., increased fidgeting, pacing). Therefore, researchers should be mindful of this effect, and ideally, attempt to monitor it as it may influence psychomotor measures. The current author recounts a presentation at a prestigious medical school involving so-called “exercise” in rodents, where a psychiatric researcher presented a protocol that involved depriving rats of their running wheels for several days - and then permitting them access to wheels to observe the effects of “exercise”. It may be more accurate to assert that such a protocol is a test of *depriving movement* as opposed to providing exercise, or perhaps both (Rhodes et al., 2003; Malisch et al., 2009; Garland et al., 2011). Such pitfalls could probably be avoided with careful design of experiments and appropriate timing of procedures.

#### To clinical practice

To reiterate, movement is a basic need and a primary drive, and loss of it may result in rapid decline of health. This is relevant for most of healthcare, but particularly true for surgery, where patients face extended time on the operating table (2+ hours) and days of bed rest. Various machines (e.g., “compression boots”) are useful to prevent some of the ill effects of inactivity (e.g., blood clots; Keith et al., 1992). Enhanced recovery protocols (e.g., ERAS) require early ambulation at regular intervals (Pędziwiatr et al., 2018). Patients who engage in this movement gain faster recovery, less pressure ulcers, shorter hospital stays, and fewer complications (Adogwa et al., 2017). There is also potential for pain analgesia and less agitation. In short, exercise is medicine (Pedersen and Saltin, 2015), and movement must be prescribed the same as the dietary regimen, pain management, and breathing/spirometry exercises.

#### To primary education

Children are most susceptible to the effects of drive, and potentially have the most to gain from movement. Classrooms should be designed to allow for greater movement, and lessons should incorporate physical activity, within reason (Bartholomew and Jowers, 2011). Sometimes small changes to lesson planning (e.g., writing, then reading) can help children to dissipate drive, while also enhancing learning (Elbow, 2004). The benefits of regular playtime and recess from studies are substantial (Ramstetter et al., 2010).

## Conclusion

The concept of drive, or motivational energy, as a precipitator of human behavior is an old but re-emerging idea - providing a unique and rarely-considered perspective on physical activity engagement. Drive is the impetus, often automatic and unconscious, to attain needs and thus restore homeostasis - returning to optimal conditions of functioning. Drive states have certain properties, as demonstrated above, such as the experience of deprivation, then tension, consumption, relief and satiation. They are under a large degree of homeostatic control but are also responsive to environmental stimuli and conditioning, including dimensions of approach and avoidance. More practically, it may be operationalized as a motivation state, characterized by negative affect, arousal, and a desire to acquire a need. Older theories of behavior highlighting drive, such as Drive Reduction Theory, have been largely sidelined in favor of higher performing models. Regardless, one may argue that movement is a primary need for most humans, most notably in younger years, but perhaps continuing well into older age. Biological processes evolved in physical bodies that moved, making these functions optimized under such conditions. As such, the need for movement can provide a significant source of tension, the relief of which is negative reinforcement – potentially strengthening the physical activity response. This tension may be measured with the CRAVE and ARGE scales, new instruments developed to evaluate desires, wants, urges, and cravings for physical activity (Stults-Kolehmainen et al., 2021b; Filgueiras et al., 2022). Conditions of being constrained or otherwise deprived of movement may result in alterations in motivation states, along with stoked feelings of being antsy, fidgety, and restless, resulting in concomitant changes in non-exercise activity thermogenesis (NEAT). However, comprehensive studies have yet to be earnestly conducted in humans. Nevertheless, this conceptualization may be useful for generating new models of behavior, such as the WANT model (Stults-Kolehmainen et al., 2020a), which will boost our understanding of movement behaviors, including physical activity and exercise.

## Data availability statement

The original contributions presented in the study are included in the article; further inquiries can be directed to the corresponding author.

## Author contributions

The author confirms being the sole contributor of this work and has approved it for publication.

## Conflict of interest

The author declares that the research was conducted in the absence of any commercial or financial relationships that could be construed as a potential conflict of interest.

## Publisher’s note

All claims expressed in this article are solely those of the authors and do not necessarily represent those of their affiliated organizations, or those of the publisher, the editors and the reviewers. Any product that may be evaluated in this article, or claim that may be made by its manufacturer, is not guaranteed or endorsed by the publisher.
